# Pericytes from Mesenchymal Stem Cells as a model for the blood-brain barrier

**DOI:** 10.1038/srep39676

**Published:** 2017-01-18

**Authors:** Xiaohe Tian, Oliver Brookes, Giuseppe Battaglia

**Affiliations:** 1 School of Life Science, Anhui University, Hefei, China; 2 Department of Chemistry, University College London, London, UK; 3 School of Engineering and Materials Science, Queen Mary University of London, London, UK

## Abstract

Blood brain-barrier (BBB) *in vitro* models have been widely reported in studies of the BBB phenotype. However, established co-culture systems involve *brain endothelial cells, astrocytes, neurons and pericytes, and therefore are often consuming* and technically challenging. Here we use mesenchymal system cells (MSC) as a potential substitute for pericytes in a BBB model. Both MSC and pericyte markers in 2D culture environment were evaluated on different extracellular matrix compositions. Further experiments indicated that MSC contributed *in a similar manner to* pericytes in a co-cultured 3D model on increasing trans-endothelial electric resistance (TEER) and decreasing permeability against macromolecules.

The blood-brain barrier (BBB) is formed from brain capillary endothelial cells joined by tight junctions (TJ), and constitutes the largest interface between circulating blood and the Central Nervous System (CNS). The BBB regulates the exchange, between blood and CNS of ions, glucose, amino acids, peptides and proteins^1^, to preserve and precisely control the CNS microenvironment for optimal function. The closely associated cells of the BBB such as astrocytes^2^, neuron cells^3^ and pericytes^4^,^5^ that collectively form the neurovascular unit (NVU) also contribute to induce characteristics of the BBB^6^,^7^. Pericytes in particular are a key component of the NVU, cells that wrap around capillaries and play crucial roles in BBB formation and regulation. They are largely responsible for many aspects of postnatal formation of BBB, including tight junction formation, and regulation of glucose transporters and vesicle trafficking amongst endothelial cells^4^,^5^. Pericytes have also been found to express smooth muscle actin (SMA), like the smooth muscle cells that adhere to the endovascular cells for blood flow regulation in microvasculature^8^. Furthermore, deficiency of pericytes has been observed to result in alteration of cerebral capillary diameter and blood flow speed^9^.

Recent work has shown strong similarities between mesenchymal stem cells (MSC) and pericytes^10^,^11^: MSCs and pericytes express many of the same cell surface markers (CD146+/CD34−/CD45−/CD56−), and CNS microvascular cells have been found to exhibit multipotential stem cell activity similar to that seen in MSCs. We hypothesize that these phenotypic similarities may translate to functional equivalence^12^. It is time consuming and technically challenging to extract and culture primary pericytes from brain tissue for BBB studies^13^. Therefore, to investigate the contribution of MSC to BBB structure and function as a potential substitute for pericytes, we here describe a BBB *in vitro* model using brain capillary endothelial cells (mouse bEND.3 cells)^14^,^15^ co-cultured with MSCs. We initially tested the expression of MSC and pericyte markers in a 2-dimentional-culture environment on different extracellular matrix compositions; we also studied the phenotypic characteristics of MSCs co-cultured with brain endothelial cells in a 3-Dimensional-Coculture environment, and the contribution to BBB characteristics such as Trans-Endothelial Electrical Resistance (TEER) and BBB permeability.

## Results

### The influence of extracellular matrix on pericyte-like behavior of MSCs

**Figure 1:**
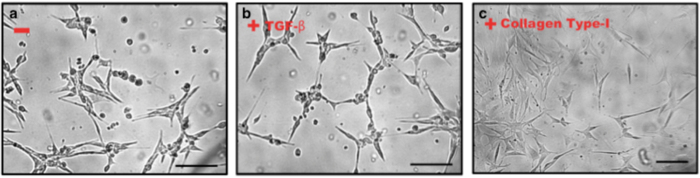
(**a**) MSC cell cultured on a plastic plate. (**b**) MSC cells cultured on a plastic plate in present of TGF- β. (**c**) MSC cell cultured on a plastic plate pre-coated rat-tail collagen (Type I).

Brain pericytes have previously been found to contribute to the up-regulation of BBB function through production and release of TGF-β (transforming growth factor)^16^,^17^. TGF-β is an important soluble mediator of communication between the endothelium and pericytes^18^; furthermore, extracellular matrix constituents such as collagen can also change pericyte morphology, marker expression and differentiation *in vitro*^19^,^20^. Here we studied the effect of TGF-β and collagen type I from the extracellular matrix on pericytes marker expression in MSCs (Mouse adult mesenchymal stem cells Gibco^®^ iMouse, C57BL⁄6). As shown in Fig. 1, mouse MSCs were cultured for up to five days on a 6-well plastic plate. To evaluate the effect of TGF-β, 200 ng/ml TGF-β was added to the medium and the medium replaced every two days. Rat-tail collagen type I was used as the extracellular matrix mimic, and pre-coated (2 μg/cm^2^) on the 6-well cell culture plate for 24 hours. Cells reached approximately 70% confluence after five days in culture. Live cells were then imaged by transmission light microscopy using a differential interference contrast (DIC) filter. As shown in Fig. 1, there was no significant change in cell morphology between non-treated cells and TGF-β treated cells (Fig. 1a,b); cell projections can be observed. In the collagen type I treated group, the cells seem to form a flatter monolayer, fewer cells detached from the culture plate, and the majority of cells showed an elongated and slender morphology (Fig. 1c). We further examined the pericyte marker expression by MSCs in these different conditions (non-treated, +TGF-β and +collagen pre-coating). Four marker genes were chosen: NG2 (NG2 proteoglycan), α-SMA (smooth muscle actin), PDGFR-β/CD140 (platelet-derived growth factor receptor-beta) and CD146 (S-endo 1-associated antigen, also referred to as MelCAM). The last is a receptor belonging to the immunoglobulin superfamily that is constitutively expressed in all human endothelial cells^21^. CD146 is also considered a marker of multi-potency for MSCs^22^.

**Figure 2:**
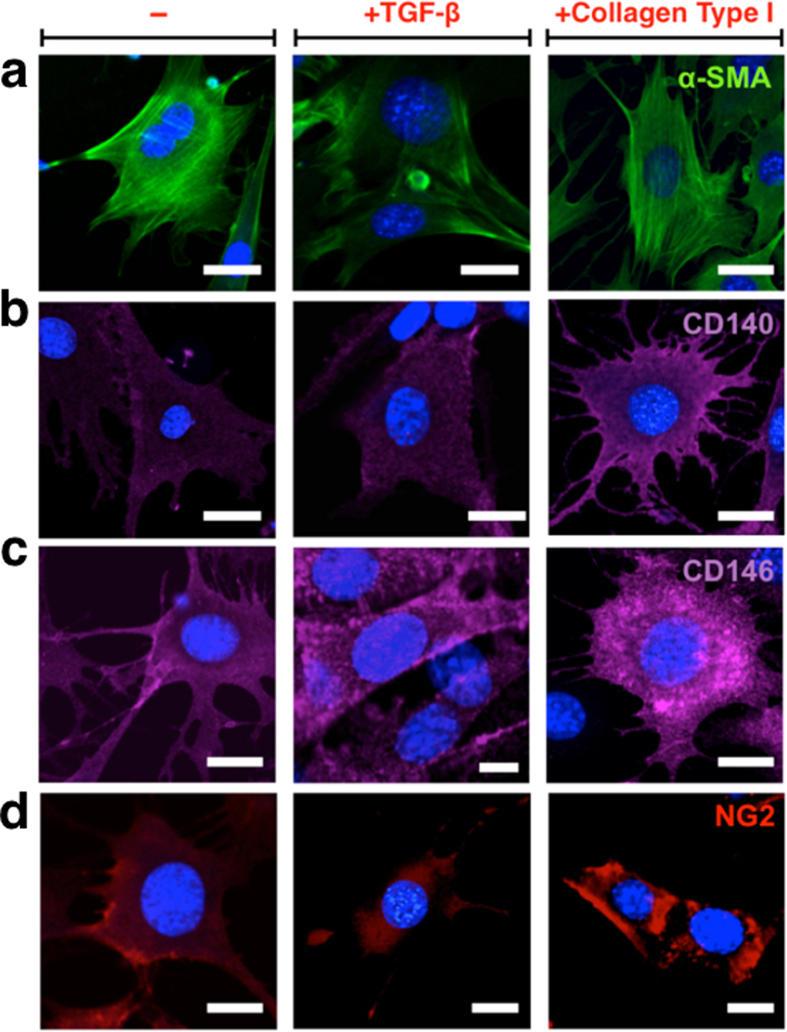
Immunofluorescence of MSC cell 2D cultured on a plastic plate with α-SMA (**a**), CD140 (**b**), CD146 (**c**) and NG (**d**) marker under non-treat, TGF-β and collagen type I conditions. Scale bars 10 μm.

It was observed that the expression of all markers increased significantly when cells were grown on a collagen I pre-coated culture plate. This condition is much closer to the *in vivo* scenario where pericytes share a basement membrane with endothelial cells on a collagen I rich extracellular matrix. The pericyte-like features of MSCs (collagen Type I coated) were further confirmed by immunofluorescence (Fig. 2). α-SMA marker clearly showed actin-fibre structure (Fig. 2a), while PDGFR-β/CD140 (Fig. 2b), CD146 (Fig. 2c) and NG2 (Fig. 2d) markers indicated distribution within the cytosol.

**Figure 3:**
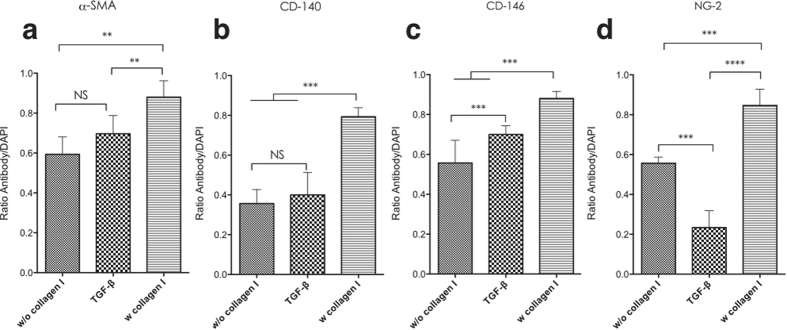
The fluorescence intensity profile from immunofluorescence of MSC cell 2D cultured on a plastic plate with α-SMA (**a**), CD140 (**b**), CD146 (**c**) and NG (**d**) marker under non-treat, TGF-β and collagen type I conditions. One-way ANOVA was used for statistical analysis, p < 0.005, Error bars: SEM.

The fluorescence intensity profile shown in Fig. 3 indicated the variation induced by TGF-β and collagen. For α-SMA and PDGFR-β/CD140 expression, there was no significant difference between non-treated cells and TGF-β treated cells, although TGF-β treated cells showed lower expression of NG2 and increased CD146 expression compare to non-treated cells. Although both non-treated and TGF-β treated cells showed positive expression of α-SMA, PDGFR-β/CD140, CD146 and NG2; the intensity was relatively lower than that observed in collagen Type I treated cells.

### TEER studies of MSCs in coculture in an *in vitro* 3D BBB model

**Figure 4:**
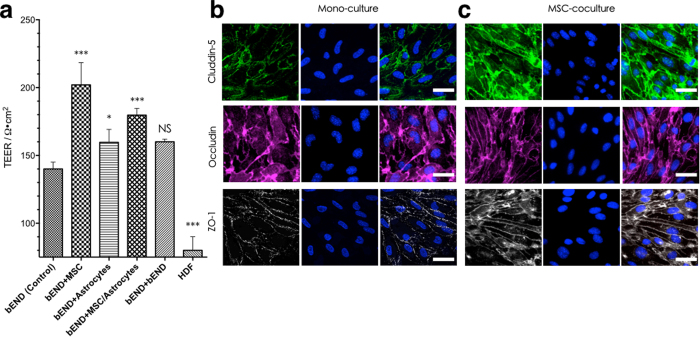
(**a**) TEER studies of MSCs in coculture BBB *in vitro* 3D model, (**b**) immunofluorescence of tight-junction expression in bEND.3 monoculture 3D model, (**c**) immunofluorescence of tight-junction expression in bEND.3/MSCs co-culture 3D model. One-way ANOVA was used for statistical analysis, p < 0.005, error bars: SEM, scale bars 20 μm.

0.4 μm filter Transwell inserts were chosen as a basis for the *in vitro* 3D BBB model. Brain endothelial cells readily form a monolayer on the filter, while the pores allow cell-cell contact with cells cultured on the obverse face. In the native BBB, the capillary endothelium is closely associated with pericytes that control many of the barrier functions of the neurovascular endothelium. To mimic more closely the BBB *in vivo,* mouse astrocytes were also added as a control. Trans-epithelial or endothelial electrical resistance (TEER) measurement was established as the most reliable, convenient and non-destructive method to quantify the integrity of endothelial monolayer or co-cultured dual-layer/multi-layer. Immortalised brain endothelial (bEND.3) cells were seeded on the transwell insert and reached 100% confluence to form a cell monolayer after seven days’ incubation. TEER was first measured at day one, and then recorded every 24 hours (day1 to day 7 TEER can be found in Supplementary Figure1) up to one week (Fig. 4a). bEND.3 cells cultured alone showed TEER continuously increasing to approximately 140 Ω.cm^2^, suggesting that permeability of the monolayer to ions diminished with increasing cell confluence. ‘Contact’ co-culture with mouse MSCs as pericytes on the opposite side of the transwell filter insert (collagen I coated) significantly raised TEER, showing that MSCs can induce tighter monolayers. bEND.3 cells co-cultured with mouse astrocytes on the underside of the filter also showed a TEER increase, to approximately 160 Ω. cm^2^, In the co-culture model with both MSCs and astrocytes underneath, the TEER was approximately 170 Ω. cm^2^ at day seven (lower than bEND.3/MSCs model but higher than bEND/astrocytes). We also performed an experiment where bEND.3 cells were cultured on both sides of the insert to test whether the simple double-layer can change TEER. As shown in Fig. 4, the TEER of such a dual-layer endothelium reaches approx. 175 Ω. cm^2^ at day seven, not significantly greater than for the single bEND.3 monolayer alone. This suggests that the barrier improvements observed with both MSCs and astrocytes are not due to a physical barrier but an actual effect on the bEND.3 cell function. This was further confirmed using a non-barrier cell, fibroblast HDF (Human Dermal Fibroblast) monolayer. This produced a TEER of approximately 90 Ω.cm^2^, much lower than that of the endothelial cell monolayer (probably due to the lack of tight junctions between the fibroblasts). Co-culture with astrocytes on the underside of the transwell filter hardly increased the TEER of the endothelial layer, this possibly due to the endothelial cells and astrocytes being separated by the 10μm-thick transwell filter, a much greater separation than is found *in vivo*. Further more, as showed in Fig. 4b,c, introduce of MSCs into 3D co-culture transwell model apparently influenced the tight junction formation and expression. Although no significant differences in individual cell morphology were observed in these two models, in monoculture condition, several cell-cell interspaces could be found; while in the bEND.3/MSCs model, the endothelial cells showed a relatively “tighter” morphology. To sum up, co-culture MSCs as pericytes clearly reduced the permeability of the model barrier, resulting in a higher TEER and more integrated tight junction morphology, suggesting that pericytes (MSCs) play a more important role in regulating the BBB properties in our culture conditions.

### MSCs act as pericytes in coculture BBB *in vitro* 3D model

**Figure 5:**
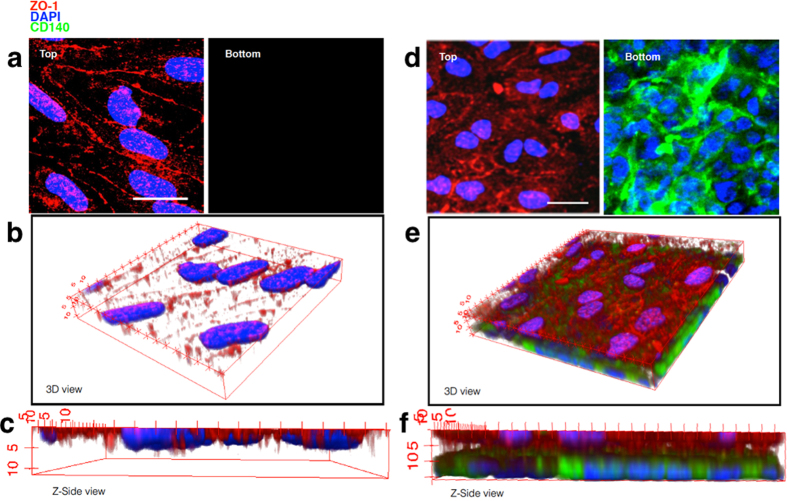
(**a**) bEND.3 monoculture 3D model with top and bottom view, 3D reconstruction view (**b**) and Z-side view (**c**). (**d**) bEND.3/MSCs co-culture 3D model with top and bottom view, 3D reconstruction view (**e**) and Z-side view (**f**). scale bars 20 μm.

TEER studies indicated that the presence of MSCs in a coculture *in vitro* model could significantly decrease the permeability of an endothelial monolayer. As shown in Fig. 5, Micrographs from the top, and bottom layers of the filter membrane respectively showed a bEND.3 monolayer. Here the brain endothelial tight junction protein ZO-1 was also stained via immunofluorescence chemistry. The whole landscape of the bEND.3 (culture) on the micro-porous filter was reconstructed within a 3D volume viewer, which clearly showed a confluent monolayer. We then tested the expression of the pericyte marker PDGFR-β/CD140 when the MSCs were introduced in the MSCs/bEND.3 coculture model, while the endothelial cell monolayer integrity was again confirmed by marking tight junctions with ZO-1. Micrographs from the top and bottom layers of the filter membrane respectively showed a bEND.3 monolayer and MSC monolayer, this “sandwich-like” composition could be viewed in the 3D projection (also refer to Movie S1). In addition, the pericyte marker α-SMA (smooth muscle actin) also showed positive expression in such BBB *in vitro* model.

### Permeability assay of the *in vitro* 3D BBB model

**Figure 6:**
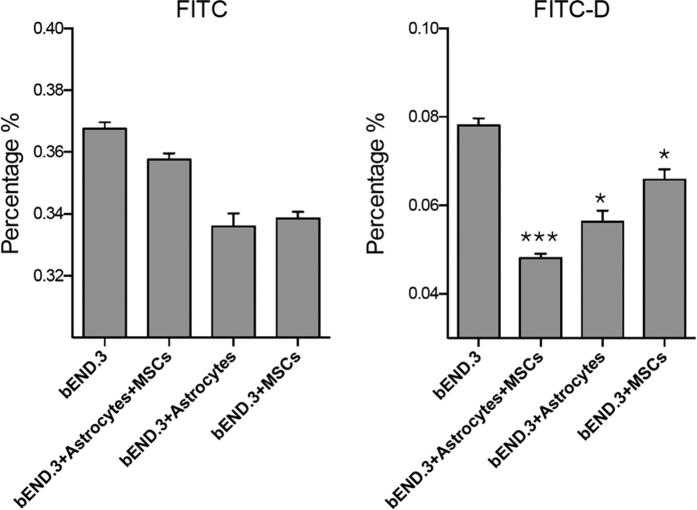
Monoculture and co-cultured transwell permeability assay for FITC and FITC-Dextran. One-way ANOVA was used for statistical analysis, n = 3, p < 0.005, error bars: SEM.

We next performed a permeability assay on such established BBB co-culture *in vitro* model. Here FITC (Mw = 389.38) and FITC labelled dextran (FITC-D, Mw~42,000) were used as the representative of small and big hydrophilic molecules. FITC and FITC-D were added in the upper compartment of the (co-cultured) transwell, the culture medium was collected 8 hours post-treatment and quantitatively measured by fluorimeter. As shown in Fig. 6, FITC crosses the *in vitro* barrier over the incubation period, and the FITC concentration on the lower section reached approximately 0.367%. There is no statically significant difference between the monoculture and the astrocyte/MSC, astrocyte and MSC co-culture models. In contrast, in FITC-D experiments, the concentration of penetrated macromolecules remains low (~ 0.008% in the bEND.3 monoculture model). It is noteworthy that at bEND.3 astrocytes/MSC co-culture model, penetrated FITC-D showed ~ 1.8 fold decrease, while astrocytes and MSC co-culture model showed ~1.3 fold decease and 1.1 fold decease respectively. This suggests that MSC coculturewas able to significantly induce the BBB impermeability to large molecules.

## Discussions and Conclusions

We showed that by using commercially available mesenchymal system cells as a substitute for pericytes, we could establish an *in vitro* model of the BBB with improved barrier characteristics. This model retained critical BBB phenotypes including expression of tight junction proteins and impermeability to macromolecules. We observed mild upregulation of different pericyte markers when cells were cultured on collagen Type-II (or Type–III) with and without growth factor. Future studies will evaluate the effect of different concentrations of TGF-β on influence on pericyte marker expression, in both 2D and 3D culture conditions. Moreover, it would be interesting to examine the effect of coculture with embryonic stem cells (ESCs) on BBB tight junction protein expression and permeability, since the current study used somatic stem cells. Finally, a more complicated 3D BBB model could be constructed by introducing astrocytes and neurons to understand the impact of using MSCs to replace pericytes in such combination. In conclusion, we demonstrated that it is possible to use MSCs in place of pericytes in co-cultured *in vitro* models of the BBB. Our results indicated that in 2D culture, MSCs expressed pericyte markers, and this can be enhanced by introducing collagen as an extracellular matrix and partly enhanced by adding TGF-β (CD-146 and NG2). Further, MSCs successfully incorporate in bEND.3/MSC and bEND.3/MSC/Astrocytes coculture models. In particular, MSC co-culture models showed barrier resistance (TEER) enhancement and blocked diffusion of a soluble macromolecule (FITC-D) across the model barrier. Our results suggest that in a well-established BBB model, MSCs may play similar roles to pericytes^23^. These cells might therefore provide a more efficient and convenient substitute for pericytes, in further BBB model research *in vitro* and *in vivo*.

## Methods

### *In vitro* 3D cell culture and assessment of barrier properties

Endothelial cells (bEND.3, ATCC^®^ CRL-2299™), were cultured in DMEM supplemented with 10% FCS, penicillin and streptomycin, L-glutamine and Fungizone. Astrocytes (ATCC^®^ CRL-2541™, C8-D1A Astrocyte Type I clone) were cultured in DMEM supplemented with 10% FCS and L-glutamine without antibiotics. Mesenchymal stem cells (MSCs) (Gibco^®^iMouse, C57BL⁄6) medium was DMEM F12 media with gluta-MAX-I, supplemented with 10% FCS and 5 μg/ml gentamicin. GIBCO^®^ Mouse (C57BL/6) Mesenchymal Stem Cells (MSCs) are produced from bone marrow isolated from C57BL/6 mice at ≤8 weeks of gestation through mechanical and enzymatic digestion. The cells were isolated under sterile conditions, expanded in D-MEM/F-12 medium containing 10% MSC-Qualified FBS, and cryopreserved at passage 8 (P8) in cryopreservation medium consisting of 60% D-MEM/F-12, 30% FBS, and 10% DMSO. For transwell experiments, both sides of the transwell insert filters (Corning^®^3460 PE filter, diameter: 1.05 cm, pore size: 0.4 μm) were pre-coated with 10 μg/cm^2^ collagen, then bEND.3 mouse brain endothelial cells were seeded on the upper surface (20,000–40,000 cells/per well) and incubated for 12 hours at 37 °C in 95% O_2_ 5% CO_2_ in order to allow the cells to fully attach. Next, the astrocytes and/or MSCs (10,000–20,000 cells/per well) were seeded on the opposite of the filter insert, and incubated for 12 hours at 37 °C in 95% O_2_ 5% CO_2_. Then the inserts were move to a transwell plate, and incubated for 7 days at 37 °C, changing the medium every two days. For TEER measurements, the cells were allowed to attach for 24–48 hours, for both monoculture and co-culture models. Resistance measurements were taken once a day with an EVOM voltohmmeter (World Precision Instruments) until cells reached confluence. The background resistance was subtracted. Unit resistance was calculated by multiplying the resistance by the area of the filter membrane (1.12 cm^2^ for 12 well-plate transwell insert), and averaged for each sample (*n* = 5).

### MSCs *In vitro* immunocytochemistry

Mesenchymal stem cells (MSCs), Gibco^®^Mouse, C57BL⁄6 were seeded in 96-well plate (BD 96-well plate, glass bottom), pre-coated with 10 μg/cm^2^ collagen (2,000–5,000 cells/per well). Cultures were maintained at 37 **°**C in an atmosphere of 5% CO_2_ and 95% air once 70–80% confluence was achieved. The cells were then were fixed with 4% formaldehyde in PBS, 15 min at room temperature. Block specimen in Blocking Buffer (1XPBS/5% normal serum/0.3% Triton ^TM^ X-100 for 60 min. Serum species may vary due to primary antibody (Ab) species. Prepare primary Ab in Antibody Dilution Buffer (1XPBS/1% BSA/0.3% Triton ^TM^ X-100), NG2 (Rabbit anti Mouse, 5 μg/ml), α-SMA (Rabbit anti Mouse, FITC directly conjugated), PDGFR-β/CD140 (Rabbit anti Mouse, 1:500), CD146 (Rabbit anti Mouse, 1:500) incubate overnight at 4 °C, or 2–4 hours at room temperature. Wash in PBS 3 times, 5 min each time. Then incubated specimen in proper fluorochrome-conjugated secondary antibody diluted in Antibody Dilution Buffer for 1–2 hrs at room temperature in the dark. Wash in PBS 3 times, 5 min each time. Then add mounting medium with DAPI. Cells were imaged on a ZEISS LSM 510 META confocal laser-scanning microscope with 63x oil immersion lens.

### Transwell *In vitro* immunocytochemistry and confocal microscopy of transwell filters

Transwell inserts were harvested after Trans-Epithelial Electric Resistance (TEER) measurements were taken with an EVOM^2^ Epithelial Voltohmmeter, and then fixed using 3.7% formaldehyde. Where immunofluorescence was performed, fixation was followed by a 30-minute incubation in 0.3% Triton X-100 and 1% bovine serum albumin (BSA). The transwell insert membrane was excised using a scalpel, and mounted on glass cover slip with VectaShield mounting medium. Cells were imaged on a ZEISS LSM 510 META confocal laser-scanning microscope and Leica SP8 confocal laser-scanning microscope with 40x water immersion lens and 63x oil immersion lens. Nuclear staining was performed using Hoechst 33342 (500 nM) for 10 min in PBS. Image data was acquired and processed using Zeiss LSM Image Browser, Zeiss LSM Image Expert, Leica and Image J software.

## Additional Information

**How to cite this article**: Tian, X. *et al*. Pericytes from Mesenchymal system cells to model for the blood-brain barrier. *Sci. Rep.* **7**, 39676; doi: 10.1038/srep39676 (2017).

**Publisher's note:** Springer Nature remains neutral with regard to jurisdictional claims in published maps and institutional affiliations.

## Supplementary Material

Supplementary Information

Supplementary Video 1
