# Supplementary material for: Pericytes from Mesenchymal Stem Cells as a model for the blood-brain barrier
Source: Sci Rep. 2017 Jan 18;7:39676. doi: 10.1038/srep39676 (PMC5241806; doi:10.1038/srep39676)
Supplement: Supplementary Information [file srep39676-s1.doc]

**Supporting Information**

**Pericytes from Mesenchymal stem cells to model for the blood-brain barrier**

Xiaohe Tian**^1, 2*^** Oliver Brookes**^3^** Giuseppe Battaglia**^2*^**

**^1^** School of Life Science, Center for Stem Cell Research and Translational Medicine. Anhui University, Hefei 230601, P. R. China. ^2^ Department of Chemistry, **^3^** School of Engineering and Materials Science, Queen Mary University of London, Mile End Road, E1 4NS, London, UK.
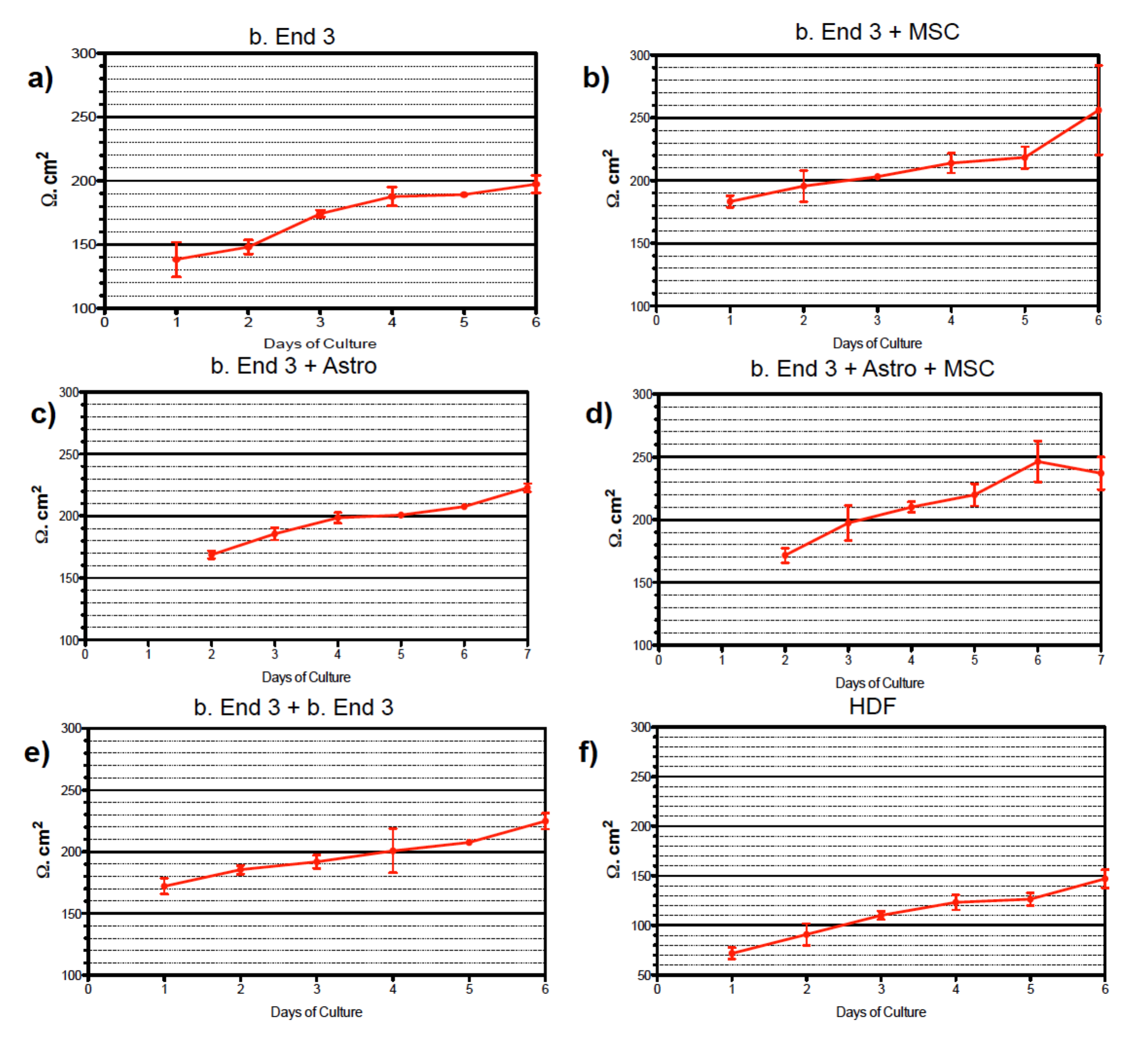


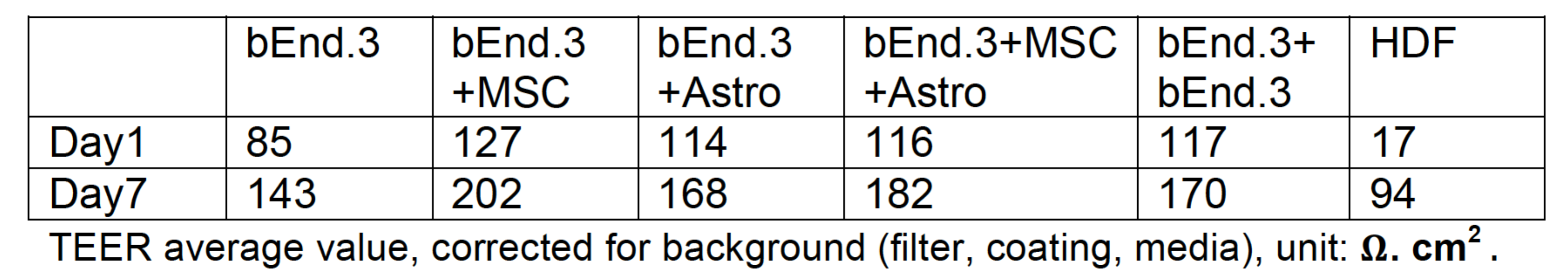


Figure S1. Transendothelial electrical resistance (TEER) of the in vitro BBB models over 7 days. a) bEND.3 cells alone. b) bEND.3 cells co-cultured with MSCs. c) bEND.3 cells co-cultured with astrocytes. d) bEND.3 cells co-cultured with astrocytes and MSCs. e) bEND.3 co-cultured with bEND.3 on the opposite side of transwell insert. f) TEER of HDF (human dermal fibroblast) cell monolayer as control group. (n=6) The tables indicate the average TEER value from all co-culture models.

Movie S1. Please refer to online supplementary video.
